# Tian Xian Liquid (TXL) induces apoptosis in HT-29 colon cancer cell *in vitro *and inhibits tumor growth *in vivo*

**DOI:** 10.1186/1749-8546-5-25

**Published:** 2010-07-21

**Authors:** Qing Liu, Yao Tong, Stephen Cho Wing Sze, Wing Keung Liu, Lam Lam, Ellie Shihng Meir Chu, Christine Miu Ngan Yow

**Affiliations:** 1 School of Chinese Medicine, Li Ka Shing Faculty of Medicine, University of Hong Kong, Pokfulam, Hong Kong SAR, China; 2 School of Biomedical Sciences, Faculty of Medicine, Chinese University of Hong Kong, Shatin, NT, Hong Kong SAR, China; 3 Medical Laboratory Science Section, Department of Health Technology and Informatics, Hong Kong Polytechnic University, Hung Hom, Hong Kong SAR, China

## Abstract

**Background:**

Tian Xian Liquid (TXL) is a Chinese medicine decoction and has been used as an anticancer dietary supplement. The present study aims to investigate the effects of TXL on the apoptosis of HT-29 cells and tumor growth *in vivo*.

**Method:**

HT-29 colon cancer cells were treated with gradient dilution of TXL. The mitochondrial membrane potential was measured by JC-1 assay. The release of cytochrome c from mitochondrial and apoptosis-related proteins *Bax*, *Bcl-2*, cleaved caspase-3, 9 were examined by Western blot analysis. HT-29 cells were implanted in nude mice to examine the effects of TXL on tumor growth.

**Result:**

TXL inhibited HT-29 xenografted model and showed a strong and dose-dependent inhibitory effect on the proliferation of HT-29 cells. Mitochondrial membrane potential was reduced by TXL at the concentration of 0.5% above. For Western blot analysis, an increase in *Bax *expression and a decrease in *Bcl-2 *expression were observed in TXL-treated cells. TXL treatment increased the protein level of cleaved casepase-3 and caspase-9, and the release of cytochrome c in cytoplasm was up-regulated as well.

**Conclusion:**

TXL significantly inhibits cell proliferation in the HT-29 cells and HT-29 xenografted model via the mitochondrial cell death pathway.

## Background

Colorectal carcinoma increased up to four folds in the past decade and the mortality is rising [1]. Much progress has been achieved in alternative medicine [2] such as Chinese medicine.

Most methods of chemotherapy for cancer induce cancer cell apoptosis. Excessive apoptosis causes hypotrophy such as ischemic damage whereas insufficient apoptosis leads to uncontrolled cell proliferation such as cancer [3]. Chemotherapeutic agents may cause mitochondrial dysfunction leading to depolarization of the inner mitochondrial membrane potential (*Δψm*) [4], triggering the caspases cascade by releasing several caspase activators. Among them, cytochrome c activates caspases by forming a complex with Apaf-1 and procaspase-9, thereby triggering caspase-9 activation which subsequently cleaves the effector caspase-3[5,6].

Tian Xian Liquid (TXL), an aqueous extraction of Chinese medicinal herbs including *Radix Ginseng, Cordyceps, Radix Astragali, Radix Glycyrrhizae, Rhizoma Dioscorea, Margarita, Fructus Lycii, Ganoderma, Fructus Ligustri Lucidi, Herba Scutellariae Barbatae*, has been used as an anticancer dietary supplement for more than a decade [7]. Previous experiments reported that TXL had inhibitory effects on human cervical carcinoma C-33A cells and human lung carcinoma H1299 cells[7]. The present study aims to investigate the effects of TXL on the apoptosis of HT-29 cells and tumor growth *in vivo*.

## Methods

### Cell culture

Human colon cancer cell HT-29 (ATCC^® ^Number:HTB-38™) was obtained from the American Type Culture Collection (ATCC, USA) and cultured in RPMI 1640 (Hyclone, USA) supplemented with fetal bovine serum (10%), penicillin (100 units/ml) and streptomycin (100 mg/ml) (Hyclone, USA) in a humidified incubator (37°C) containing 95% air and 5% CO_2_. Trypsin (Hyclone, USA) was used for trypsination.

### Preparation of TXL

Tian Xian Liquid (TXL) (Batch number: L2-171040) was provided by China-Japan Feida Union Company Ltd. and stored away from light at 4°C. TXL was diluted and incorporated into the cell culture medium RPMI 1640. Residues were removed by filtration.

### Cell proliferation

Cell proliferation was assessed *in vitro *with 3-(4,5-Dimethylthiazol-2-yl)-2,5-Diphenyltetrazolium Bromide (MTT) according to the manufacturer's protocol (Roche, USA). HT-29 cells (10000 per well) were incubated in triplicates in a 96-well plate. TXL was serially diluted with RPMI1640 and the final concentrations were 0.25, 0.5, 1, 2 and 5%. The plates were incubated with or without TXL for 24 and 48 hours. At the end of the incubation, cells were exposed to MTT (10 μL, 5 mg/mL in phosphate-buffered saline) in culture medium for four hours at 37°C. The supernatant was removed and 150 μL DMSO (Sigma, USA) was added to dissolve the formazan crystals. The absorbance was measured at 595 nm with an ELISA plate reader (Bio-Rad, USA).

### DAPI staining

DAPI (Sigma, USA) (4' 6-diamidino 2-phenylindole)-stained nuclei were observed with fluorescence microscopy. HT-29 cells (70-80% confluent) in 24-well uncoated plates were exposed to 0.5% and 1% TXL for 24 hours respectively. Cells were fixed with 4% paraformaldehyde for 30 minutes and incubated with 1 μg/mL DAPI solution for 30 minutes in the dark. Stained cells were imaged under a fluorescence microscope (Carl Zeiss, Germany).

### Assessment of apoptosis by determination of mitochondrial membrane potential

Mitochondrial membrane potential was assessed by 5, 5', 6, 6'-tetrachloro-1, 1', 3, 3'tetraethylbenzimidazolylcarbocyanine iodide (JC-1) according to the manufacturer's protocol (Biotium, USA). After trypsinization and centrifugation (500× *g*)(Eppendorf, Germany) for ten minutes at room temperature, the pellets of cell culture with or without TXL were re-suspended in RPMI 1640 medium (1 ml), stained with 5 mg/ml JC-1 for 30 minutes at 37°C in the dark, washed twice in phosphate buffered saline (PBS) and re-suspended in 0.5 ml PBS. *Δψm *depletion was observed under a fluorescence microscope. A green filter was used for green-fluorescent monomer at depolarized membrane potentials and a red filter for orange-fluorescent J-aggregate at hyperpolarized membrane potentials.

To measure the quantitative change of mitochondrial potential, we applied JC-1 with fluorescence plate reader. Briefly, cells (1 × 10^5^) in 100 μl culture medium/well were seeded in black 96-well plate (Nunc, Denmark) and treated with TXL (0.15, 0.3, 0.6, 1.25 and 2.5%). After 24 and 48 hours incubation, JC-1 (5 μg/ml) was added for the last 30 minutes of treatment. Cells were washed twice with PBS to remove unbound dye. The concentration of retained JC-1 dye was measured (490 nm excitation/600 nm emission) with a luminescence spectrometer (PerkinElmer, USA).

### Western blot

The HT29 cells were incubated with increasing concentrations of TXL (0, 0.5%, 0.75%, 1%) for 48 hours. For the time-course experiment, HT29 were treated with 1% TXL for 12, 24, or 48 hours. Cellular levels of cleaved caspase-3, 9 (Cell Signaling Technology, USA) *Bax*/*Bcl-2 *cytochrome C and glyceraldehyde 3-phosphate dehydrogenase (GAPDH) (Santa Cruz Biotechnology, USA) were determined by Western blot. Lysates were prepared from 1 × 10^7 ^cells by dissolving cell pellets in 100 μl of lysis buffer. Lysates were centrifuged (Eppendorf, Germany) at 18000× *g *for 15 minutes and the supernatant was collected. The protein concentration was estimated with the Bio-Rad protein assay kit (Bio-Rad, USA) using bovine serum albumin as a standard. Sample proteins were resolved by 10% sodium dodecylsulfate polyacrylamide gel (Bio-Rad, USA) electrophoresis and then electrophoretically transferred to PVDF membrane (Millipore, USA) and blocked with 5% BSA (Sigma, USA). Subsequently the primary antibodies caspase-9, cleaved caspase3, *Bax*, *Bcl-2*, cytochrome C and GAPDH were added. After overnight incubation at 4°C the blots were washed, exposed to HRP-conjugated corresponding secondary antibodies for one hour and finally were visualized by ECL Advanced Solution (GE Healthcare Life Sciences, USA). Digital images were captured by Gel Doc™ gel documentation system (Bio-Rad, USA) and intensity was quantified using Quantity-One software version 4.62(Bio-Rad, USA).

### In vivo tumor-growth inhibition studies

The experiment was approved by the Department of Health, Hong Kong SAR, China and the Committee on the Use of Live Animals in Teaching and Research (CULATR) of Li Ka Shing Faculty of Medicine, University of Hong Kong. Six-week-old female nude mice were purchased from the Laboratory Animal Unit, University of Hong Kong and kept under sterile conditions in accordance with the institutional guidelines of animal care. The HT-29 carcinoma was established in nude mice by injecting the suspensions of HT-29 (1 × 10^6 ^cells per animal) [8] cells subcutaneously into the right flank of each animal. When the tumors became palpable (size: 18 mm3) after xenografting, mice were divided into three groups (n = 8) by a random numbered table: (1) Control group orally administered with 200 μl PBS); (2) 5-fluorouracil (5-FU) (Choongwae, Korea) group (injected intraperitoneally with 5-FU, 30 mg per kg of body weight) three times a week [9,10]; (3) TXL group (orally administered with 200 μl TXL daily for 14 days. To evaluate the antitumor activity of TXL, we measured the tumor volume with a digital caliper six times every week (from day1 to day 6 and from day 8 to day 14) and calculated using the formula: (longest diameter) × (shortest diameter)^2 ^× 0.5. The body weights of all animals were recorded throughout the experiment to assess drug toxicity.

### Statistical analysis

Data were presented as mean and standard deviation (SD). When one-way ANOVA showed significant differences among groups, Tukey's *post hoc *test was used to determine the specific pairs of groups that were statistically different. A level of *P *< 0.05 was considered statistically significant. Analysis was performed with the software SPSS version 16.0 (SPSS Inc, USA).

## Results

### Anti-proliferative and apoptotic effects of TXL on HT-29 cells

**Figure 1:**
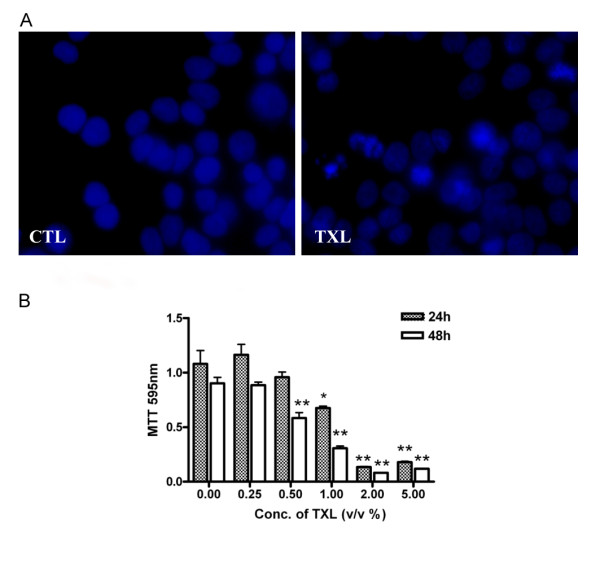
**TXL's inhibitory effects on cellular growth and apoptosis in HT-29 cells**. (A) Apoptosis in HT-29 after TXL treatment for 48 hours was determined by staining the cell with DAPI. Apoptotic cell exhibiting characteristic chromatin condensation were observed by fluorescence microscopy. (B) Cell proliferation was assessed after 24 and 48 hours with the MTT assay as described in Methods. Results (optical densities) are expressed as mean and standard deviation (*n *= 3). The experiment was repeated three times with similar results. CTL: control.

To investigate the anti-proliferative effects of TXL on HT-29 cells, we treated the HT-29 cells with TXL in a gradient of doses (0.25-5%) and cell proliferation after two days was assessed with the MTT assay in triplicates. The results were consistent. TXL inhibited HT-29 cell proliferation in a dose-dependent manner (Figure 1). Treatment of TXL (1%) for 48 hour significantly inhibited (38.47%; *P *< 0.05, *P *= 0.011) cell proliferation.

### Effects of TXL on cell nuclear morphology

Nuclear staining with DAPI was used to determine apoptosis-inducing activity of TXL in HT-29 cells. After TXL (1%) treatment, HT-29 cells underwent typical morphologic changes of apoptosis including nuclear condensation and formation of apoptosis bodies (Figure 1).

### Treatment of TXL reduces the mitochondrial membrane potential

**Figure 2:**
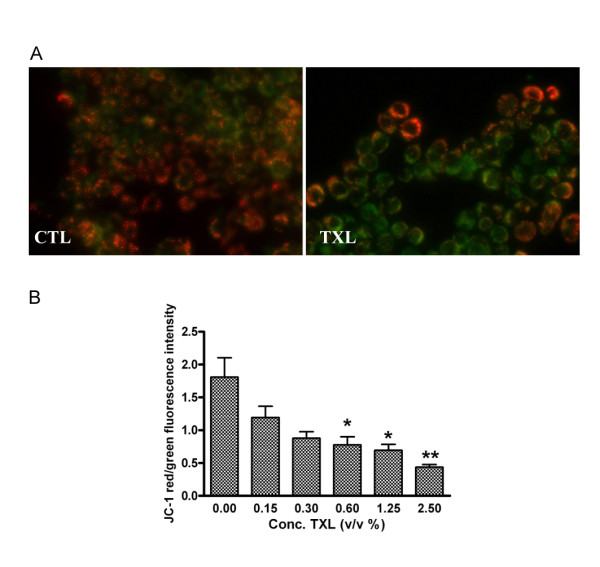
**TXL's effect on the depolarization of HT-29 mitochondria**. (A) JC-1 staining observed by fluorescence microscopy. TXL treated cells showed a majority of cells stained green dye due to low mitochondrial membrane potential. (B) Effect of TXL on the depolarization of HT-29 mitochondria was also measured by fluorescence plate reader using JC-1. The cells were exposed to increasing concentrations of TXL. Data represent mean and standard deviation of three individuals with asterisks denoting significant differences between controls and TXL-exposed cells (*P < 0.05, **P < 0.01). CTL: control.

JC-1, a cationic dye, produces red fluorescent J-aggregates in mitochondria with high *Δψm *and green fluorescence with low *Δψm*. Most control cells had red J-aggregation fluorescence whereas TXL-treated cells had green fluorescence (Figure 2A). JC-1 staining was used to determine mitochondrial integrity. To quantify the change of mitochondrial potential, we applied JC-1 with fluorescence plate reader. The green to red fluorescence ratio significantly decreased at 48 hours in a dose-dependent manner (Figure 2B).

### TXL triggers interaction between Bcl-2 and Bax and releases cytochrome c

**Figure 3:**
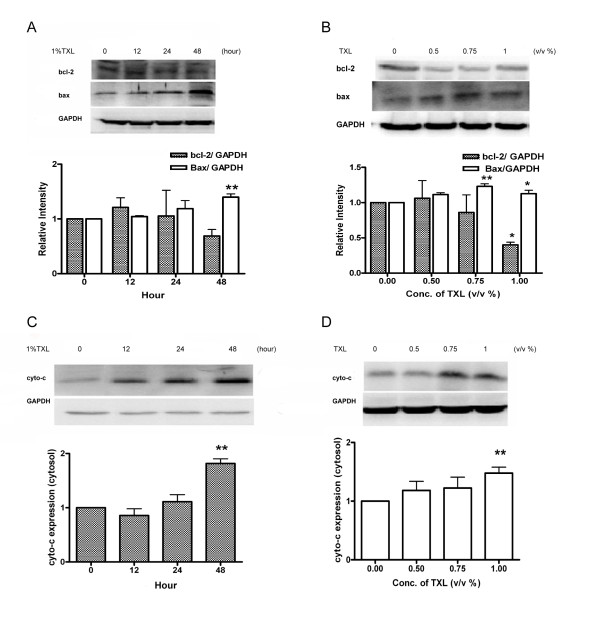
**Effect of TXL on cytochrome c**. Total protein from HT-29 treated with 1%TXL for 12, 24 and 48 hours (A) or indicated concentration of TXL for 48 hours (B) were analyzed by Western blot with specific antibodies against *Bax *and *Bcl-2*. Protein from cytosolic fraction of HT-29 which has been treated with TXL (1%) for 12, 24 and 48 hours (C) or indicated concentration of TXL for 48 hours (D) were analyzed by Western blot with specific antibodies against cytochrome c. GAPDH antibody was used as control for equal loading. The relative expressions of proteins were quantified using Bio-Rad Quatity-One software. Results are expressed as mean and standard deviation (*n *= 3),*P < 0.05 **P < 0.01compared with control group.

Low *Δψm *is regulated by *Bcl-2 *family proteins [11]. We studied the effects of TXL on the expression of *Bax *and *Bcl-2 *which are important for mitochondrial membrane permeablization. In this study, the HT-29 cells were incubated with increasing concentrations of TXL (0, 0.5%, 0.75%, 1%) for 48 hours. For the time-course experiment, HT29 were treated with 1% TXL for 12, 24, or 48 h. Cell lysates were prepared for western blot analysis. After 48 hours, HT-29 cells treated with 1% TXL showed significant up-regulation (*P  *= 0.003) in *Bax *expression (Figure 3) while significant down-regulation (*P  *= 0.013) in *Bcl-2 *expression (Figure 3). TXL (0.75%) and TXL (1%) increased the *Bax*/*Bcl-2 *ratio by 1.4 and 2.8 folds respectively in HT-29 cells. Stability of mitochondrial membrane is influenced by the interactions among *Bcl-2 *family proteins, thereby affecting the release of cytochrome c from mitochondria to and subsequently accumulation in the cytosol [12]. The cytosol levels of cytochrome c in HT-29 cells were examined with Western blot. In HT-29 cells treated with TXL, cytochrome c significantly increased in a dose-dependent (*P *= 0.0096) and time-dependent manner (*P  *= 0.001).

### TXL induces caspase-3 and caspase-9 cleavage

**Figure 4:**
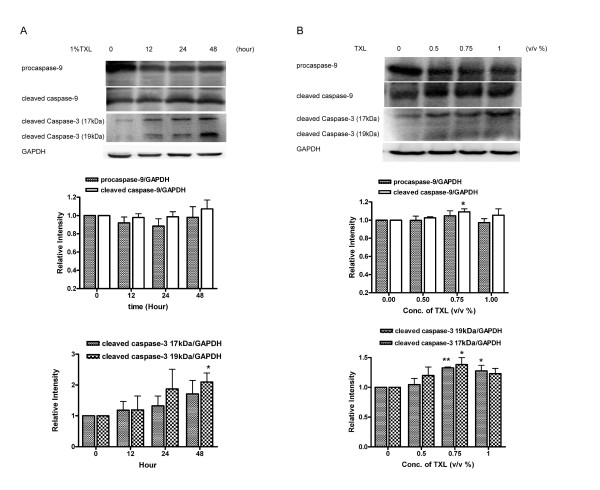
**TXL Induces caspase-3 and caspase-9 cleavage**. Protein from HT-29 which has been treated with 1%TXL for 12, 24 and 48 hours (A) and protein from HT-29 which has been treated with indicated concentration of TXL for 48 hours (B) were analyzed by Western blot with specific antibodies against caspase-9 and cleaved caspase-3. GAPDH antibody was used as control for equal loading. The relative expressions of proteins were quantified using Bio-Rad Quatity-One software. Results are expressed as means and standard deviation (*n *= 3),*P < 0.05 **P < 0.01 compared with control group.

To confirm the induction of the mitochondrial-mediated apoptosis, we examined the activation of the intrinsic initiator caspase-9 and casapse-3 using western blot. TXL (0.75% and 1%) induced the cleavage of caspase-3 to its active form, i.e. p17 (17 kDa) which was found after 24 hours of TXL treatment (Figure 4). As shown in Figure 4, caspases-9 in HT29 cells treated with TXL was activated, as judged by the decrease of the procaspases-9 and the increase of their cleavage products.

### In vivo effects of TXL on HT-29 tumor growth

**Figure 5:**
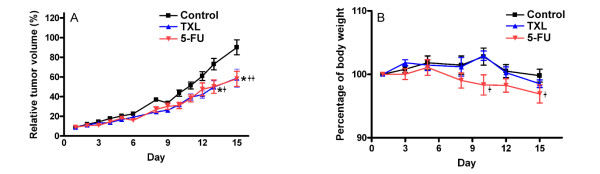
**Effect of TXL on tumor volume (A) and body weight (B) in HT-29-bearing mouse model**. HT-29 cells were injected in nude mice as described in Methods. A total of 24 mice were divided into three groups. Data are expressed as mean and standard deviation (*n *= 8). Significantly difference compared with control group:*^+ ^*P *< 0.05, **++ *P *< 0.01.

To determine the antitumor efficacy of TXL as a single agent therapy, we examined the growth of HT-29 cells in immunocompromised mice. Compared with mice orally administrated with 200 μl PBS as control group, treatment with the TXL and 5-FU significantly inhibited tumor growth (Figure 5A). After treatment of nude mice with TXL, the tumor size was significantly (*P  *= 0.03) decreased from day 13 to day 15. The difference in tumor size in the TXL group (*P *= 0.933) was not significant compared with the 5-FU group (*P *= 0.99).

## Discussion

Most chemotherapeutic drugs induce cancer cell apoptosis whereby a cell activates its own destruction by initiating a series of cascading events including the loss of the mitochondrial transmembrane potential [6]. A rapid collapse of mitochondrial transmembrane electrical potential Δ*ψ*_m _is always found in chemotherapeutic agents-induced apoptosis in cancer cells [13]. The present study demonstrated that TXL-induced apoptosis was related to the collapse of the mitochondrial membrane potential Δ*ψ*_m_.

Our study showed the depletion of *Δψm *(Figure 2) and the activation of caspase-3 of HT-29 treated with TXL. Mitochondria participate in apoptosis induction by releasing several caspase activators. Among them, cytochrome c activates caspases by forming a complex with Apaf-1 and procaspase-9, thereby triggering caspase-9 activation which subsequently cleaves the effector caspase-3 [6]. The present study found that 1% of TXL induced the cleavage of caspase-3 to its active form, namely p17 (Figure 4). The fragment, p17 (17 kDa), was accumulated after 24 hours of TXL treatment. In this study, we also observed that TXL remarkably increased the release of cytochrome c from the mitochondria to the cytosol in HT-29 cells. Levels of cytochrome c in the cytosolic fraction increased dramatically when the dosage of TXL was 0.5% or above. These results suggest a direct link between the mitochondria and the TXL-induced apoptosis.

A previous study showed that mitochondrial membrane disruption and the release of cytochrome c was controlled by *Bcl-2 *family protein [11]. *Bcl-2 *and other pro-apoptotic factors prevent mitochondrial membrane disruption while *Bax *promotes these events. To clarify whether *Bcl-2 *family was changed in TXL treated HT-29 cell to activate the release of cytochrome c, we examined the expression level of *Bcl-2 *and *Bax *with or without the TXL treatment. An increasing *Bax *and a decreasing *Bcl-2 *were observed in a time-dependent manner after exposed to 1% TXL. Our results showed that TXL induced apoptosis by increasing the *Bax/Bcl-2 *ratios. These observations confirmed that TXL induced apoptosis in colon cancer via the mitochondrial pathway. The above concomitant molecular events in TXL-treated HT-29 cells result in remarkable apoptosis process. Further *in vivo *and *in vitro *studies are needed to clarify the protein interactions, thereby delineating the upstream regulatory events, such as the Wnt signaling pathway which is important factor in the development of the majority of colorectal cancers[14].

We studied TXL's effects on the growth of HT-29 cell lines grown *in vitro *and compared those results with its effect on tumor growth *in vivo*. We found that TXL attenuated the growth of xenografted HT-29 tumors *in vivo*. The injection of 5-fluorouracil (5-FU), a common choice for single-agent chemotherapy of advanced colon cancer [15,16] on nude mice significantly attenuated tumor growth (Figure 5A). However, during the 5-FU treatment, the body weight of nude mice was significantly decreased on day 10 (*P *< 0.05, *P *= 0.038) (Figure 5B). The components of TXL such as *Radix Ginseng*, *Cordyceps*, *Radix Astragali*, *Fructus Lycii*, *Ganoderma *are commonly used in China as immune-stimulating agents. Whether TXL's immunomodulation effect and protection effect on stomach and digestive system reducing toxic side effects of 5-FU will be investigated in the future.

Because 5-fluorouracil (5-FU) as common choice for single-agent chemotherapy of advanced colon cancer is also recognized for its toxicity including fatigue, diarrhea and sometimes myelosuppression, much attention has been focused on exploring complementary and alternative medicine. This study may provide a platform for evaluation the function of Chinese medicine decoctions on treatment of cancer, which has no significant side effect. To further evaluate the potential of TXL as an adjuvant agent in colon cancer chemotherapy, we are studying TXL's effect on attenuation of 5-FU-induced side effect and the synergistic anti-tumor effect of the 5-FU/TXL.

## Conclusion

TXL significantly inhibits cell proliferation in the HT-29 cells and HT-29-bearing mouse model. TXL-induced apoptosis is likely achieved through the mitochondrial cell death pathway as indicated by a reduction in mitochondrial membrane potential, and the decrease of *Bcl-2*/*Bax *ratio and the release of cytochrome c followed by the activation of caspase-3 and caspase-9.

## Abbreviations

TXL: Tian Xian Liquid; 5-FU: 5-fluorouracil; *Δψm: *mitochondrial membrane potential; MTT: 3-(4,5-Dimethylthiazol-2-yl)-2,5-Diphenyltetrazolium Bromide; DAPI: 4' 6-diamidino 2-phenylindole; JC-1: 5, 5', 6, 6'-tetrachloro-1, 1', 3, 3'tetraethylbenzimidazolylcarbocyanine iodide; PBS: phosphate buffered saline; SD: standard deviation; GAPDH: glyceraldehyde 3-phosphate dehydrogenase.

## Competing interests

This research has received a grant from China-Japan Feida Union Company Ltd.

## Authors' contributions

QL performed the experiments, analyzed data and drafted the manuscript. YT and SCWS designed the study and revised the manuscript. ESMC and LL conducted the *in vivo *experiments. WKL designed the *in vivo *experiment and prepared the human colon cancer cells. CMNY designed the *in vitro *experiment. All authors read and approved the final manuscript.
